# Supplementary material for: From sequence to enzyme mechanism using multi-label machine learning
Source: BMC Bioinformatics. 2014 May 19;15:150. doi: 10.1186/1471-2105-15-150 (PMC4229970; doi:10.1186/1471-2105-15-150)
Supplement: Additional file 2 — Java code of ml2db. Additional file ml2db_code.tar.gz contains the Java source code to run the multi-label machine learning experiments and save the results to database. The code’s Javadoc is included. [file 1471-2105-15-150-S2.zip › additional file 2/ml2db/ml2db/doc/mulan/classifier/InvalidDataException.html]

InvalidDataException


JavaScript is disabled on your browser.


- Overview
- Package
- Class
- Use
- Tree
- Deprecated
- Index
- Help

- Prev Class
- Next Class

- Frames
- No Frames

- All Classes

- Summary:
- Nested |
- Field |
- Constr |
- Method

- Detail:
- Field |
- Constr |
- Method


mulan.classifier

## Class InvalidDataException

- java.lang.Object
- - java.lang.Throwable
  - - java.lang.Exception
    - - java.lang.RuntimeException
      - - mulan.core.MulanRuntimeException
        - - mulan.classifier.LearnerException
          - - mulan.classifier.InvalidDataException

- All Implemented Interfaces:
  :   java.io.Serializable

  ---

    

  ```
  public class InvalidDataException
  extends LearnerException
  ```

  Exception thrown by the `MultiLabelLearner` when presented with invalid data or
  data which can not be processed by the specific learner type.

  Author:
  :   Jozef Vilcek

  See Also:
  :   Serialized Form

- - ### Constructor Summary

    Constructors

    Constructor and Description

    `InvalidDataException(java.lang.String message)`

    Creates a new instance of `InvalidDataException` with the specified
    detail message.

    `InvalidDataException(java.lang.String message,
    java.lang.Throwable cause)`

    Creates a new instance of `InvalidDataException` with the specified
    detail message and nested exception.
  - ### Method Summary

    - ### Methods inherited from class java.lang.Throwable

      `addSuppressed, fillInStackTrace, getCause, getLocalizedMessage, getMessage, getStackTrace, getSuppressed, initCause, printStackTrace, printStackTrace, printStackTrace, setStackTrace, toString`
    - ### Methods inherited from class java.lang.Object

      `equals, getClass, hashCode, notify, notifyAll, wait, wait, wait`

- - ### Constructor Detail


    - #### InvalidDataException

      ```
      public InvalidDataException(java.lang.String message)
      ```

      Creates a new instance of `InvalidDataException` with the specified
      detail message.

      Parameters:
      :   `message` - the detail message


    - #### InvalidDataException

      ```
      public InvalidDataException(java.lang.String message,
                          java.lang.Throwable cause)
      ```

      Creates a new instance of `InvalidDataException` with the specified
      detail message and nested exception.

      Parameters:
      :   `message` - the detail message
      :   `cause` - the nested exception


- Overview
- Package
- Class
- Use
- Tree
- Deprecated
- Index
- Help

- Prev Class
- Next Class

- Frames
- No Frames

- All Classes

- Summary:
- Nested |
- Field |
- Constr |
- Method

- Detail:
- Field |
- Constr |
- Method
